# Cyclophilin A: An Independent Prognostic Factor for Survival in Patients with Metastatic Colorectal Cancer Treated with Bevacizumab and Chemotherapy

**DOI:** 10.3390/cancers16020385

**Published:** 2024-01-16

**Authors:** Diana Cornelia Moisuc, Daniela Constantinescu, Mihai Vasile Marinca, Bogdan Gafton, Mariana Pavel-Tanasa, Petru Cianga

**Affiliations:** 1 Immunology Department, “Grigore T. Popa” University of Medicine and Pharmacy, 700115 Iasi, Romania; cornelia-diana-i-miron@d.umfiasi.ro (D.C.M.); d.constantinescu@umfiasi.ro (D.C.); 2 Immunology Department, “St. Spiridon” Hospital, 700111 Iasi, Romania; 3 Oncology Department, “Grigore T. Popa” University of Medicine and Pharmacy, 700115 Iasi, Romania; mihai.marinca@umfiasi.ro (M.V.M.); bogdan.gafton@umfiasi.ro (B.G.); 4 Oncology Department, Regional Institute of Oncology, 700483 Iasi, Romania

**Keywords:** cyclophilin A, prognostic factor, bevacizumab, chemotherapy, metastatic colorectal cancer

## Abstract

**Simple Summary:**

Colorectal cancer ranks as the second most common cause of cancer-related deaths. In this research, we analyzed the prognostic and predictive roles of three potential serum biomarkers (Cyclophilin A (CypA), copeptin and Tie2) in 56 patients with metastatic colorectal cancer treated with bevacizumab and chemotherapy. We assessed the serum levels of biomarkers before and after one and six months of therapy. Based on Kaplan–Meier curves and multivariate Cox analysis, low levels of CypA at baseline and after one month of treatment were associated with better overall survival, being independent prognostic factors after adjustment for baseline and post-treatment factors. For copeptin and Tie2, the results show no correlation between these biomarkers and overall survival or progression-free survival.

**Abstract:**

Colorectal cancer (CRC) ranks as second most common cause of cancer-related deaths. The CRC management considerably improved in recent years, especially due to biological therapies such as bevacizumab. The lack of predictive or prognostic biomarkers remains one of the major disadvantages of using bevacizumab in the CRC management. We performed a prospective study to analyze the prognostic and predictive roles of three potential serum biomarkers (Cyclophilin A (CypA), copeptin and Tie2) investigated by ELISA in 56 patients with metastatic CRC undergoing bevacizumab and chemotherapy between May 2019 and September 2021 at baseline and after one and six months of therapy. We showed that low levels of CypA at baseline and after one month of treatment were associated with better overall survival (OS) (42 versus 24 months, *p* = 0.029 at baseline; 42 versus 25 months, *p* = 0.039 after one month). For copeptin and Tie2, Kaplan–Meier curves showed no correlation between these biomarkers and OS or progression-free survival. When adjusting for baseline and post-treatment factors, a multivariate Cox analysis showed that low values of CypA at baseline and after one month of treatment were independent prognostic factors for OS and correlated with a better prognosis in metastatic CRC patients.

## 1. Introduction

Colorectal cancer (CRC) is cited as the third most frequent malignancy and second leading cause of cancer-related deaths worldwide [1]. If diagnosed at an early stage, CRC is correlated with a favorable prognosis, but 15–30% of patients already have metastatic disease at the time of diagnosis. Among patients diagnosed during the initial stages, approximately 50% will gradually progress to metastatic disease [2]. The management of CRC has significantly improved in recent years and survival rates are increasing. Although several factors have thus far been acknowledged to contribute to an improved clinical outcome, a significant role is attributed to new biologic therapies that target either the epidermal growth factor (EGFR) or the vascular endothelial growth factor (VEGF). Angiogenesis plays an important role in the proliferation and dissemination of solid tumors. The inhibition of angiogenesis with molecules that directly target VEGF, or the extracellular or intracellular portion of its receptors, has become standard treatment for several types of neoplasia, including CRC.

Bevacizumab is a humanized monoclonal IgG antibody that binds to VEGF-A and inhibits angiogenesis by preventing the interaction of VEGF-A with VEGFR1 and VEGFR2 [3]. Nonetheless, following the use of targeted therapies to inhibit angiogenesis, a new toxicity profile has emerged. The most common adverse effects were hypertension, increased bleeding risk, thromboembolic events, development of proteinuria, fatigue, fistulas, impaired wound healing and heart failure [3]. Pooled data from retrospective analyses show that bevacizumab-induced hypertension appears to be linked with a substantial enhancement in the response rate and disease progression time, leading to the hypothesis that bevacizumab-related hypertension could be considered a predictive clinical biomarker. A meta-analysis of seven studies investigating the efficacy of bevacizumab showed that patients who experienced an onset of hypertension during treatment with bevacizumab had an improvement in the survival and response rates [4].

Numerous pathways contribute to CRC carcinogenesis, and the thorough characterization of its phenotypic and molecular aspects is an important step in determining prognosis and treatment approaches. Currently, personalized medicine is a growing topic of discussion and involves tailoring treatment strategies for each patient according to the specific characteristics of the tumor, genetics, environment, and lifestyle. Key aspects of personalized medicine include genomic profiling, targeted therapies, and the identification of predictive or prognostic biomarkers [5]. The identification of a clinical or molecular biomarker with predictive or prognostic value is an important aspect. Although bevacizumab improves overall survival (OS) and progression-free survival (PFS), not all patients clinically benefit from it. Identifying those particular patients is difficult because there are still no predictive biomarkers for antiangiogenic therapy. VEGF was the most studied biomarker in clinical trials [6,7], but the results obtained from these studies are contradictory.

Cyclophilin A (CypA) is an intracellular protein that plays a role in the pathogenesis of atherosclerosis, hypertension and angiogenesis. It is secreted by macrophages, endothelial cells and vascular smooth muscle cells due to inflammatory and oxidative stimuli [8]. Secreted CypA mediates intercellular signaling and activates pro-inflammatory signaling in endothelial and vascular smooth muscle cells. CypA participates in various processes during cancer development. CypA overexpression has been reported to stimulate cell proliferation, migration and invasion, as well as inhibiting apoptosis [8,9,10]. CypA might enhance the survival of malignant cells, playing an important role in preserving the conformation of oncogenic and signaling proteins involved in carcinogenesis. These proteins are highly expressed in various cancer cell types, such as small cell lung cancer, pancreatic cancer, breast cancer and colorectal cancer [11]. Moreover, CypA expression was shown to be related to angiogenesis and the proliferation of endothelial cells derived from the human umbilical vein [12]. Hypoxia is prevalent in solid tumors, leading to the frequent generation of reactive oxygen species. It has been suggested that CypA may exert antioxidant effects through its peptidyl–prolyl cis-trans isomerase activity. The regulation of CypA expression is controlled by p53 and the hypoxia-inducible factor 1-alpha (HIF 1α) [11]. These factors activate the transcription of genes involved in angiogenesis and the resistance to hypoxic circumstances. HIF 1α also participates in cellular metabolism by promoting glycolysis and inhibiting mitochondrial respiration. In response to hypoxia, CypA expression increases in malignant tumors [13].

Vasopressin is a peptide that exhibits instability and is characterized by a short half-life of 15–20 min [14]. Copeptin is a surrogate marker of vasopressin secretion. An elevated level of copeptin is associated with a poor prognosis in sepsis, stroke, pneumonia, myocardial ischemia and diabetes [15,16]. Elevated levels of the three circulating vasoactive peptides—Mid-Regional Pro-Adrenomedullin, Mid-Regional Pro-Atrial Natriuretic Peptide and C-terminal-prepro-vasopressin (copeptin)—in CRC patients who received bevacizumab therapy are associated with an enhancement in progression time, indicating a potential association between the tumor vascularization and the patient’s circulatory system [17]. In addition to the effect on vascular tone, these peptides also appear to play a role in angiogenesis [18]. Vasopressin stimulates the synthesis of endothelial cell proteins and endothelin 1, a vasoactive peptide that stimulates the secretion of VEGF in vascular smooth muscle cells [19].

Tyrosine kinase with immunoglobulin and epidermal growth factor homology domains 2 (Tie2) is the receptor for angiopoietins. This receptor is expressed in vascular endothelial cells, where it controls their survival, multiplication, migration, adhesion and spreading. It is also expressed in tumor-associated macrophages and tumor cells and is known to influence various biological processes, such as neovascularization and cell adhesion to the extracellular matrix [20,21]. The Tie2 ligands are Angiopoietin 1 (Ang-1) and 2 (Ang-2) [20]. Studies show that Tie2 plays an important role in angiogenesis [21,22,23]. In cervical tumors, Tie2-high tumor cells stimulate the upregulation of Tie2 and VEGFR2 expression on endothelial cells through the activation of the Tie2-AKT/MAPK signaling pathway, thereby facilitating the angiogenesis process [21]. Other studies show that Ang-1 and Tie2 levels are predictive biomarkers for better PFS in patients with ovarian cancer who are treated with bevacizumab [22], and that plasma levels of Ang-1, Ang-2 and Tie2 can be considered colorectal cancer biomarkers [23].

The objective of this prospective study was to identify suitable biomarkers that might help in properly selecting the metastatic colorectal cancer patient’s subgroup that will benefit from treatment with bevacizumab and chemotherapy. We hypothesized that some of the established markers for angiogenesis should also be useful for predicting the sensitivity of mCRC to treatment with bevacizumab plus chemotherapy, in terms of tumor response. To achieve this goal, we analyzed and compared the prognostic and predictive role of three serum biomarkers—CypA, copeptin and Tie2—in patients with metastatic CRC undergoing treatment with bevacizumab and chemotherapy. Our results show that, among the three investigated biomarkers, serum CypA values, both at baseline and after one month of treatment, best predict the efficacy of bevacizumab therapy.

## 2. Materials and Methods

### 2.1. Patients

Our study included a cohort of metastatic CRC patients who received bevacizumab and chemotherapy between May 2019 and September 2021 at the Regional Institute of Oncology, Iasi. 

Patient inclusion criteria were as follows: (1) age over 18 years old; (2) Eastern Cooperative Oncology Group 0–2 performance status; (3) life expectancy of at least 6 months; and (4) normal hematological, renal and hepatic function at diagnosis. Exclusion criteria were as follows: (1) known hypersensitivity to bevacizumab or chemotherapy; (2) acute ischemic disease; (3) moderate heart failure; and (4) any other condition that might affect patient’s compliance (e.g., psychiatric pathologies).

The treatment regimen received by our patients followed institutional and international guidelines, including 7.5 mg/kg of bevacizumab every 3 weeks or 5 mg/kg every 2 weeks and chemotherapy established by the attending physician: oxaliplatin-based chemotherapy (*n* = 37), irinotecan-based chemotherapy (*n* = 16) or fluoropyrimidine-based chemotherapy (*n* = 3). The options for associated chemotherapy regimens were as follows: CapeOX (oxaliplatin 130 mg/m^2^ intravenous (iv) infusion on day 1 and oral capecitabine 1000 mg/m^2^ twice daily on days 1–14, every 3 weeks), mFOLFOX6 (oxaliplatin 85 mg/m^2^ iv, fluorouracil 400 mg/m^2^ iv bolus and 2400 mg/m^2^ continuous iv infusion for 46 h, folinic acid 400 mg/m^2^ iv administration on day 1, every 2 weeks), XELIRI (irinotecan 250 mg/m^2^ iv infusion on day 1 and oral capecitabine 1000 mg/m^2^ twice daily on days 1–14, every 3 weeks), FOLFIRI (irinotecan 185 mg/m^2^ iv, fluorouracil 400 mg/m^2^ iv bolus and 2400 mg/m^2^ continuous iv infusion for 46 h, folinic acid 400 mg/m^2^ iv administration on day 1, every 2 weeks), oral capecitabine monotherapy 1000 mg/m^2^ twice daily on days 1–14, every 3 weeks, or de Gramont regimen (fluorouracil 400 mg/m^2^ iv bolus and 2400 mg/m^2^ continuous iv infusion for 46 h, folinic acid 400 mg/m^2^ iv administration on day 1, every 2 weeks). 

Patient and disease characteristics are summarized in Table 1.

The study was approved by the institutional Ethics Committee (Regional Institute of Oncology, Iasi) and informed consent was obtained from all participants.

### 2.2. Specimen Characteristics

Fasting blood was collected on the day when the initial course of bevacizumab and chemotherapy was administered, one month later and then 6 months after the initiation of therapy. Blood samples were collected in vacutainers with no anticoagulant and processed within 6 h of being received. More precisely, blood was spun at 2000 G for 5 min, and aliquots of 500 μL of the separated serum were stored at −80 °C until further analysis of biomarker profiles. 

### 2.3. Assay Methods

The biomarker profiles were assessed at the Immunology Laboratory of St Spiridon County Emergency Hospital, Iasi. Specific enzyme-linked immunosorbent assay kits were used to quantify the serum levels of CypA (Elabscience Sandwich-ELISA kit, E-EL-H6096, Wuhan, China), copeptin (Elabscience Competitive-ELISA kit, E-EL-H0851, Wuhan, China) and Tie2 (Abbexa Sandwich-ELISA kit, abx153297, Cambridge, UK) according to the manufacturer’s instructions. The ELISA plates were read for absorbance at 450 nm using the TECAN reader infinite 200 pro. The assays were performed while being blinded to the study endpoint.

### 2.4. Study Design

We performed a prospective study in on a cohort of 56 CRC patients who had been diagnosed with metastatic-stage cancer and received bevacizumab and chemotherapy between May 2019 and September 2021. From this cohort of 56 patients, 44 were diagnosed with de novo metastatic CRC in the specified time frame (May 2019–September 2021), while 12 patients previously diagnosed with localized CRC (between June 2015–May 2019), were included in our research since they had developed metastases. The median time from cancer diagnosis to bevacizumab-based therapy was 5 months (range: 1–67 months). From these 56 cases, 21 patients were still alive at the time of the analysis (May 2023), and the longest registered overall survival (OS, defined here as the amount of time from diagnosis to death) was 96 months.

This was a single-arm cohort study. The primary endpoint compared the overall survival between patients with high and low levels of the biomarkers, categorized based on the median value for each measurement. Secondary endpoints included progression-free survival (PFS, referred here as the amount of time from the initiation of bevacizumab therapy until disease progression or death), tumor response rate, and the presence of adverse events. The treating physician evaluated the tumor response after 6 months of treatment, through computed tomography, magnetic resonance or positron emission tomography following the revised RECIST guidelines (version 1.1) [24], and the results were interpreted as complete response (RC), partial response (PR), stable disease (SD) and progressive disease (PD). The treatment-related adverse events (AEs) were additionally monitored and classified according to the Common Terminology Criteria for Adverse Events v4.0, including the renal (the onset of proteinuria, assessed by the summary urine test and defined as the presence of proteins over the cut-off level of 30 mg/dL), hematological (anemia, neutropenia, thrombocytopenia, assessed by the analysis of complete blood count (CBC)), neurological, and liver (assessed by the serum levels of alanine-aminotransferase (ALT) aspartate-aminotransferase (AST), gamma-glutamyl transpeptidase (GGT)) toxicities. Other variables included in our models were as follows: baseline factors, such as CEA (low versus high, cut-off: 5 ng/mL); CA 19-9 (low versus high, cut-off: 30 U/mL); LDH (low versus high, cut-off: 246 U/L); the number of regional lymph node invasions (none, one to three, and more than three); the number and sites of metastases; and resection of the primary tumor status.

Finally, the relationships between the levels of serum biomarkers (CypA, copeptin, Tie2) and the above-mentioned clinical and paraclinical outcomes were explored.

### 2.5. Statistical Analysis

Statistical analyses were performed using the statistical software program SPSS v.20.0 (IBM SPSS, Armonk, NY, USA) and Graph Pad Prism, v5 (Graph Pad Software, San Diego, CA, USA). The patient’s description group and disease characteristics are presented as frequency, median and interquartile range (IQR, for non-parametric variables) or mean and standard deviation (for normally distributed data). OS and PFS were evaluated using Kaplan–Meier curves and the Log-rank test was used to compare distinct groups of patients. The multivariate Cox analysis was performed to evaluate the role of CypA, with OS being used as a dependent variable. The other variables included were as follows: various baseline factors (CEA, CA 19-9, LDH), the number of regional lymph node invasions (none, one to three, and more than three), the number and sites of metastases, and resection of the primary tumor and post-treatment factors: anemia, proteinuria, thrombocytopenia, neutropenia and liver toxicity. As this was a cohort study, the sample size was not defined upfront. 

The REMARK checklist for this study, as recommended by the EQUATOR (Enhancing the QUAlity and Transparency Of health Research) Network guidelines [25], is detailed in the Supplementary Table S1. 

## 3. Results

### 3.1. Patient and Disease Characteristics

This study included a cohort of 56 metastatic CRC patients who received bevacizumab and chemotherapy between May 2019 and September 2021. From the 56 CRC patients included in the study, 44 were diagnosed de novo in the metastatic stage. The other 12 patients, initially diagnosed in a non-metastatic stage, received adjuvant chemotherapy and were included in our research only when metastases were detected. The median time from cancer diagnosis to bevacizumab-based therapy was 5 months (range: 1–67 months). The study group was gender-balanced; the male to female ratio was 1.15. The median age of the patients was 61 years (range: 37–82 years). The most frequent location of the primary tumor was on the descending colon (73%). Liver metastases were the most frequent (44%), and 24 patients had multiple metastatic sites. Mutations in RAS (KRAS, NRAS) genes were present in 36 cases; three patients were not tested. 

The primary tumor was resected in 40 patients. From the entire cohort, only 5 patients underwent radiotherapy. The most commonly associated chemotherapy regimen was oxaliplatin-based (administered in 66% of cases). Pre-existing arterial hypertension was present in 48% of cases, but it was medically controlled at the time of bevacizumab treatment initiation, although 71% of patients presented episodes of high blood pressure during the treatment. The most common therapeutic classes used for treating hypertension were beta-blockers and diuretics. The response rate was 23.2% and the disease control rate was 67.8%. The baseline patient and disease characteristics are summarized in Table 1.

### 3.2. Adverse Events

The safety profile of bevacizumab and chemotherapy for our study group is described in Table 2. Proteinuria (53.6%) and neurological toxicity (44.7%) were the most common AEs regardless of grade. Among grade 3 and 4 AEs, the most frequent were neutropenia (12.5%) and anemia (9%). There was only one grade 4 AE (neutropenia). No cases of grade 5 AE were recorded.

### 3.3. Serum Biomarkers

**Figure 1:**
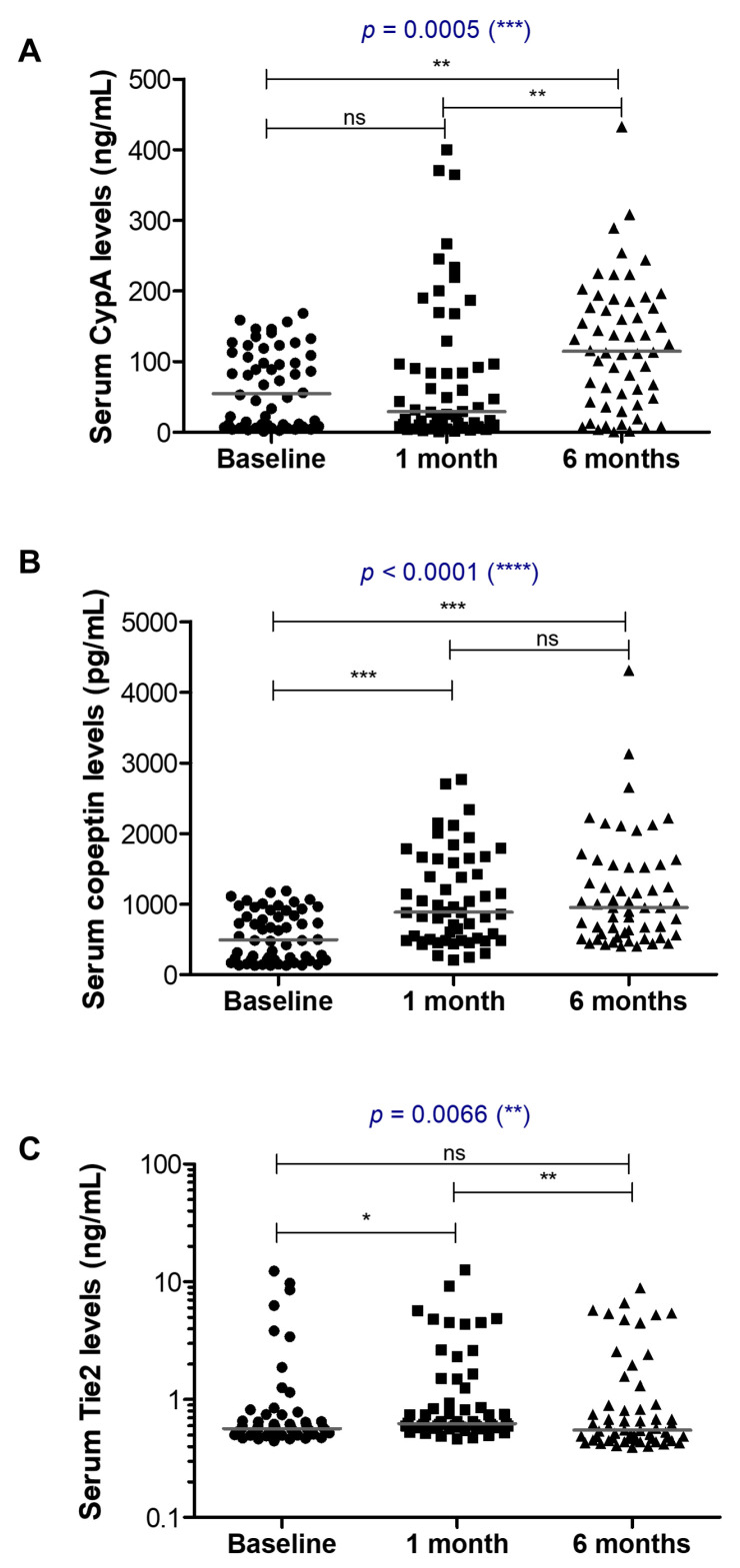
Serum levels of the indicated biomarkers at baseline, and after one month or six months of treatment. (**A**) Cyclophilin A, (**B**) Copeptin, and (**C**) Tie2 (**** *p* < 0.0001, *** *p* < 0.001, ** *p* < 0.01, * *p* < 0.05, ns—not significant; Kruskal–Wallis followed by Dunn’s Multiple Comparison tests). The horizontal grey line indicates the median value for each group of cases.

The distribution and evolution of the serum biomarkers (CypA, copeptin and Tie2) are shown in Figure 1, and serum concentration and median values are shown in Table 3. Importantly, we noticed a general increasing trend for CypA (Figure 1A) and copeptin (Figure 1B) from baseline to 6 months of treatment, suggesting the efficacy of the bevacizumab treatment. Tie2 did not follow a similar pattern, showing an initial increase at 1 month of treatment, but dropped to baseline values after 6 months (Figure 1C).

To investigate the association between these serum markers and OS in patients with metastatic CRC, we next assessed the prognostic potential of CypA, copeptin and Tie2 using Kaplan–Meier curves and the Log Rank test. The median OS for the cohort was 32 months (range: 12–96 months) and the median PFS was 10 months (range: 6–36 months).

#### 3.3.1. CypA

**Figure 2:**
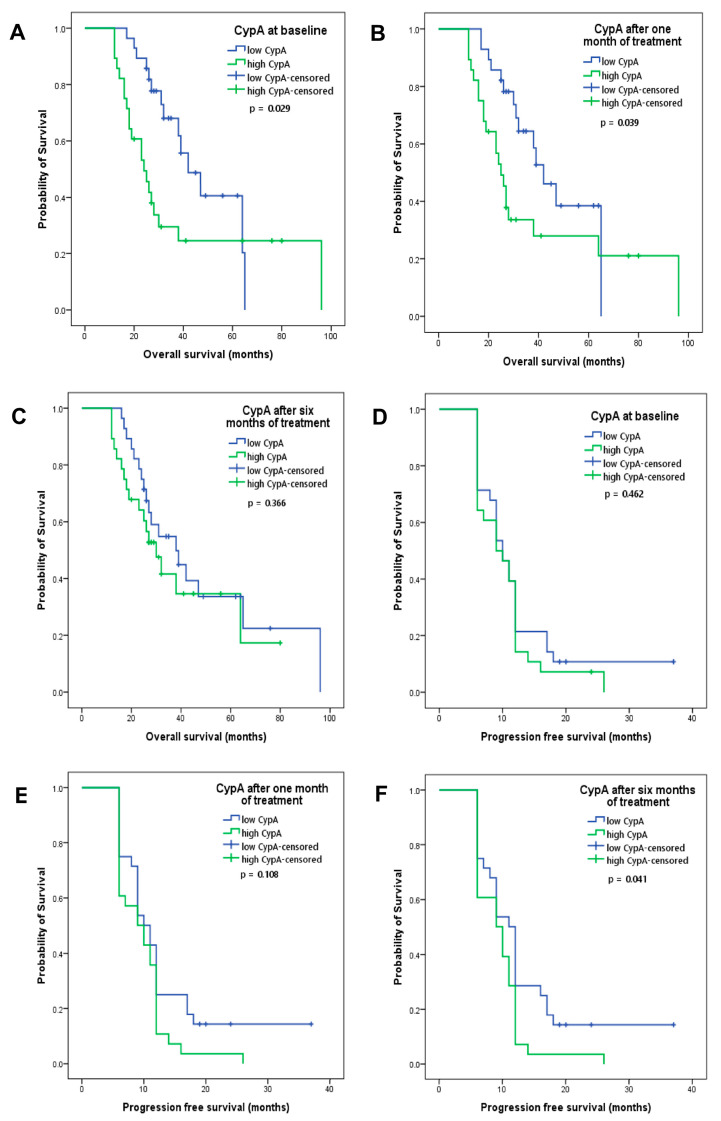
Kaplan–Meier curves of overall survival for patients with low or high CypA values at (**A**) baseline, (**B**) after one month and (**C**) after six months of treatment. Kaplan–Meier curves of progression-free survival for patients with low or high CypA values at (**D**) baseline, (**E**) after one month and (**F**) after six months of treatment.

Kaplan–Meier curves for CypA at baseline showed that low levels (median of 54.65 ng/mL) were associated with better OS (*p* = 0.029, (Figure 2A). The median OS for low levels of CypA at baseline was 42 months; for high levels of CypA, it was 24 months. After one month of treatment, the association between low values of CypA (lower than the median of 29.38 ng/mL) and OS was maintained (42 versus 25 months, *p* = 0.039; Figure 2B). After 6 months of treatment, a tendency to associate low CypA (lower than the median of 114.92 ng/mL) values with better OS was observed but without statistical significance (38 versus 30 months, *p* = 0.366; Figure 2C). In terms of PFS, while CypA values, both at baseline and after one month of treatment, did not correlate with PFS (Figure 2D,E), low CypA values were associated with better PFS only after 6 months of treatment, (11 versus 9 months, *p* = 0.041; Figure 2F). Overall, these data indicate that CypA values at baseline and after 1 month of treatment may be used in predicting OS and CypA values after 6 months of treatment may be used in predicting PFS.

#### 3.3.2. Copeptin and Tie2

For copeptin and Tie2, the Kaplan–Meier curves showed no correlation between these biomarkers and OS or PFS (Supplementary Figure S1—copeptin; Supplementary Figure S2—Tie2).

### 3.4. Clinical and Paraclinical Characteristics as Prognostic Factors

**Figure 3:**
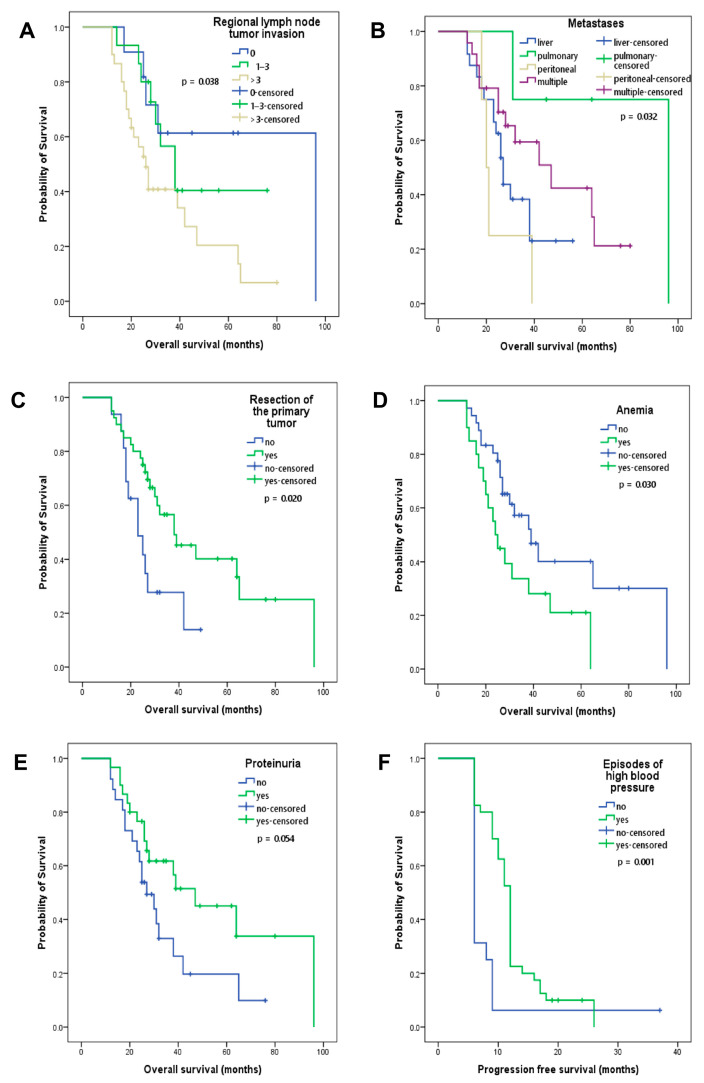
Kaplan–Meier curves of overall survival depending on (**A**) regional lymph node tumor invasion, (**B**) location of metastases, (**C**) resection of the primary tumor, (**D**) treatment-induced anemia, (**E**) proteinuria and (**F**) Kaplan–Meier curves of progression-free survival for patients who presented episodes of high blood pressure during treatment.

The number of invaded regional lymph nodes, classified according to the TNM staging, was associated with OS. Patients without regional lymph node tumor invasion presented better OS compared to those with more than three invaded lymph nodes (96 versus 26 months, *p* = 0.038; Figure 3A). Patients with peritoneal metastases had the shortest OS compared to those with liver, multiple or lung metastases (20 versus 27 versus 47 versus 96 months, *p* = 0.032; Figure 3B). Resection of the primary tumor was associated with better OS (38 versus 23 months, *p* = 0.020; Figure 3C), which is explained by an early diagnosis and the benefit of adjuvant chemotherapy. Treatment-induced anemia was associated with lower OS, regardless of grade (39 versus 24 months, *p* = 0.030; Figure 3D). Patients who presented proteinuria (53%) had better OS, but without statistical significance (47 versus 27 months, *p* = 0.054; Figure 3E). Patients who had episodes of high blood pressure during treatment had a significantly better PFS (12 versus 6 months, *p* = 0.001, Figure 3F) and an improved OS; however, this was not statistically significant (38 versus 27 months, *p* = 0.344). Among these investigated clinical and paraclinical factors, the lack of regional lymph node tumor invasion, resection of the primary tumor, the absence of anemic episodes and the presence of proteinuria proved to be indicative of better OS.

### 3.5. CypA at Baseline and after One Month of Treatment as Prognostic Factors

Next, we aimed to evaluate if the association of the CypA at baseline and after one month of treatment with OS is independent of clinical and paraclinical co-variates; therefore, a multivariate Cox analysis was performed (Table 4). Each assessment was adjusted according to baseline factors (CEA, CA 19-9, LDH), the number of invaded regional lymph nodes, the number of metastases, the resection of the primary tumor and post-treatment factors: anemia, proteinuria, thrombocytopenia, neutropenia, liver toxicity. Low values of CypA at baseline proved to be prognostic factors for a better OS when adjusting for baseline and post-treatment factors, while the low values of CypA after one month of treatment were prognostic markers for better OS when adjusting only for the post-treatment factors. Low values of CypA after 6 months of treatment were predictive of a better PFS when adjusting for treatment-induced hypertension (*p* = 0.01, HR = 0.461, 95% CI 0.253–0.840; Table 5). These data suggest that adjusting for number of lymph node invasions, anemia, proteinuria or hypertension may improve the prognostic capacity of CypA at baseline or after one month of treatment for OS or PFS.

## 4. Discussion

Antiangiogenic therapy that inhibits VEGF is largely used in oncology, including for colorectal cancers, but its practical utilization is hindered due to the absence of useful biomarkers as indicators of clinical utility. Currently, multiple VEGF inhibitors are available, which help to improve the survival of most cancer patients, but some patients gain little or simply no benefit from their administration due to resistance to these therapies. Although intensive efforts have been made to identify biomarkers that could enhance treatment optimization with VEGF inhibitors, predictive or prognostic biomarkers have proven elusive. In this study, we explored a panel of three serum biomarkers in 56 patients with metastatic CRC who were undergoing bevacizumab and chemotherapy at baseline and after one and six months of treatment. The results show that lower values of CypA, both at baseline and after one month of treatment, represent a prognostic factor, being associated with a better OS regardless of the adjusted clinical or paraclinical factors. After 6 months of treatment, low CypA values were associated with better PFS. Little is known about CypA in colorectal cancer and even less about the relationship between CypA and bevacizumab therapy. To the best of our knowledge, this is the first study that investigates the role of this marker in colorectal cancer patients undergoing treatment with bevacizumab and chemotherapy.

Through cellular signaling cascades cancer, the interaction between CypA and the cellular receptor CD147 stimulates cell proliferation [10], migration and invasion [26], metastasis [8] and, ultimately, drug resistance [27]. CypA was overexpressed in H446 cells, which originate from a small cell lung cancer [28]. Exogenous CypA protein can induce the dose- and time-dependent proliferation of H446 cells by stimulating protein kinase 1/2 (ERK1/2) signaling. In hepatocellular carcinoma (HCC), CypA induces cell proliferation by promoting the cell cycle transition from the G1 to the S phase. Furthermore, CypA was significantly higher in stage III and IV compared with stage I and II HCC, being associated with the TNM stage [29]. Xu et al. studied the expression and signaling mechanism of CD147 in 40 CRC cases and four cell lines. Their results showed that CD147 expression was increased in 62.5% CRC samples [26]. In addition, statistical analyses indicated that CD147 expression was associated with lymph node metastasis, promoting the invasion and migration of CRC cells. The loss of E-cadherin is a frequent characteristic of epithelial-to-mesenchymal transition (EMT). The overexpression of CD147 decreased the expression of the epithelial marker E-cadherin and upregulated the mesenchymal marker vimentin, suggesting that CD147 plays a role in the regulation of EMT in CRC cells. In contrast, the inhibition of CypA in colorectal cancer cells leads to decreased migration and invasion through the inhibition of EMT induction and the activation of the p38 signaling pathway, without any impact on cellular proliferation [30]. During EMT, cells downregulate their E-cadherins, thus losing their epithelial features; in contrast, they gain mesenchymal characteristics by upregulating N-cadherins [26,30]. Guo et al. showed that CypA promotes lung cancer metastases by activating p38 MAPK. The results from studies using mouse models show that the number of tumor metastases is higher in the CypA-overexpressed group [31]. In contrast, although the expression level of CypA as increased in 63% of gastric adenocarcinoma samples compared to matched samples of normal mucosa, the recurrence and metastasis rate was lower in patients with positive CypA immunostaining after 3 years of follow-up [32]. Kim et al. studied the association between CypA and angiogenesis [12], and they found that the stimulation of human umbilical vein endothelial cells with exogenously expressed CypA had a direct effect on endothelial cells in vitro. At low concentrations, CypA increased endothelial cell growth, migration, invasiveness and tubulogenesis. At high concentrations, CypA had opposite effects, decreasing endothelial cell migration and viability. In a mouse model designed for experimental atherosclerosis research, the level of CypA was evaluated in the endothelial cells of neocapillaries of carotid artery lesions, and the results show that there is an association between CypA and pathological angiogenesis. Another study showed that the expression of CD147 is associated with that of VEGF in acute myeloid leukemia. The expression of the two factors increased in patients with a high level of microvessel density and correlated with bone marrow microvessel density. The simultaneous expression of both CD147 and VEGF in bone marrow indicated a poor prognosis [33]. Only a few studies address the correlation between CypA and CD147. In one such study, Sakamoto et al. explored CD147 and CypA expression in cutaneous T-cell lymphomas and reported that the values were overexpressed by tumor cells as compared to normal skin [34]. Furthermore, they were able to show that the CypA values correlated with the level of expression of the CypA receptor, which further correlated with the disease severity. Another study evaluated CD147 and CypA in the kidneys of patients with COVID-19 and concluded that their expression was increased in tubular epithelia, as well as in podocytes and parietal cells [35]. Thus, the body of literature available suggests that the levels of CypA and CD147 may be correlated.

Another study evaluated the level of CypA in tumor tissues in colon cancer using qRT-PCR analysis [36]. The mRNA expression levels exhibited a significant increase in cancer compared to non-cancerous regions. The immunohistochemical results also show that the CypA expression level was significantly higher in cancer regions. Peng et al. studied the function of CypA in modulating the oxidative status in cancer cells and CRC chemoresistance through redox modifications [27]. They found that CypA protein levels were overexpressed in chemoresistant CRC cell lines compared to parental cells. Furthermore, they studied a cohort of 49 CRC cases divided into two arms: responders and non-responders to FOLFOX. An immunohistochemistry analysis showed increased levels of CypA in the CRC tissues from the group that did not respond to treatment. This study also revealed that levels of CypA and peroxiredoxin 2 were positively correlated in the CRC tissues. After analyzing the GEO and TCGA databases, the researchers showed that increased values of CypA were correlated with the overall survival rate in patients with CRC, indicating the prognostic value of CypA. The results of our study are consistent with these results. Patients with values lower than the median at the initiation of bevacizumab and chemotherapy had a better OS, 42 versus 24 months (42 versus 25 months after one month of treatment).

An explanation for this unfavorable prognosis is that CypA may lead to chemoresistance. Choi et al. showed that CypA upregulation could induce drug resistance by suppressing the generation of reactive oxygen species and inhibiting mitochondrial membrane potential depolarization. CypA expression is mediated by HIF 1α, which regulates cell responses to hypoxia. Upregulated CypA decreases the apoptosis induced by hypoxia and cisplatin [37]. They used human cervical carcinoma HeLa, human prostate carcinoma DU145 and human colorectal carcinoma HCT116 p53+/+ and p53−/− cells treated with and without cisplatin, which are known to induce apoptosis by producing reactive oxygen species. Their results are confirmed by another study showing that CypA mediates colorectal cancer chemoresistance through redox modification [27].

According to Chen et al., another mechanism is found in the high levels of CypA that enhance the expression of genes associated with drug resistance, such as multidrug resistance-associated protein 2, IL-6 and glutathione S-transferase zeta 1, [38]. Their results indicate that elevated CypA leads to doxorubicin and vincristine resistance in SK-Hep1 cell lines in drug sensitivity and drug accumulation assays, suggesting that CypA may facilitate tumor cells to survive, even in unfavorable environments such as hypoxia and chemotherapy. Furthermore, the stimulation of CypA/CD147 signaling induces radiotherapy resistance in hepatocellular carcinoma through the interaction with integrin β1 and activation of the downstream FAK/PI3K-Akt pathway. The inhibition of CD147 increased the radiosensitivity of hepatocellular carcinoma cells, and the CD147 blockade decreased the proliferation and metastatic potential of tumor cells [39].

As we were interested in understanding if CypA levels impact OS in patients treated only with chemotherapy, we searched the GEO database for cohorts of CRC patients for whom CypA protein expression levels were assessed. We discovered the GEO:GSE17536 database, which has been publicly available since 2009 and includes gene expression profiles that predict recurrence and mortality in colon cancer patients. Out of an initial cohort of 177 patients, we excluded patients with CRC stage I and II because they did not undergo chemotherapy, and further analyzed OS depending on CypA expression levels in the tumor tissue. The results show that CypA did not influence OS, and the difference between the two groups (low versus high, depending on the median value) was not statistically significant (*p* = 0.492). In another study, Peng et al. stated that the CypA protein levels negatively correlated with the overall survival rate in CRC patients from the GEO: GSE17536 database [27]. However, their approach was different, in the sense that they included all 177 patients and the cut-off value for CypA was not mentioned. To further verify these results, we also analyzed another database of gene expression profiling, including CypA, in 185 ovarian tumors: GSE26712. We did not find any statistically significant association between OS and CypA protein levels, not even when we divided the study group based on the extremely low values of CypA (below the 25th percentile). Corroborating the results of our research and those obtained from various database analyses, we concluded that low CypA values are prognostic factors for bevacizumab therapy, rather than prognostic factors for systemic chemotherapy in colorectal cancers. This could only be confirmed by studies evaluating the administration of bevacizumab without chemotherapy, but bevacizumab monotherapy is outside the therapeutic standards of CRC. Therefore, we cannot exclude the notion that CypA levels do not influence chemotherapy resistance. However, several other factors may influence chemotherapy resistance. Some studies indicate that microRNAs, which are short, endogenous, single-stranded, non-coding RNAs, usually 18–25 nucleotides long and capable of post-transcriptionally suppressing gene expression, play a role in the development of chemoresistance in CRC [5,40]. The overexpression of microRNA-21 dramatically reduces the therapeutic efficacy of 5-FU by diminishing the activity of the essential protein complex involved in mismatch repair recognition: human mutS homologs 2 and 6 [40]. In addition, elevated microRNA-203 levels show a correlation with resistance to oxaliplatin, while the dysregulation of other miRNAs, such as microRNA-10b, has been linked to resistance against 5-fluorouracil and irinotecan [5].

Taken together, all these data show that serum CypA plays a significant role in the pathogenesis of colorectal cancer, representing a potential negative prognostic factor for CRC patients treated with bevacizumab and chemotherapy, but also a promising target for advancing the cancer treatment strategies.

In our study, neither copeptin nor Tie2 correlated with OS or PFS. In another study, potential correlations between circulating levels of vasoactive peptides, including copeptin, and tumor responses while undergoing bevacizumab therapy in CRC patients were investigated. Contrary to our results, the results of that study show that increased levels of vasoactive peptides, including copeptin, from baseline and after six weeks of treatment were correlated with better outcomes. The same study also investigated a possible association between vasoactive peptide levels and hypertension, but no connection was identified [17]. The results of a recent study show that the levels of copeptin correlate with cardiac toxicity in patients with colorectal or anal cancer who underwent chemotherapy with 5-FU. Serum copeptin levels were shown to significantly increase after the first 5-FU cycle and progressively increase during subsequent 5-FU cycles [41]. It is known that bevacizumab causes cardiotoxicity, clinically manifested by cardiovascular side effects such as decreased left ventricular ejection fraction, vasculitis, hypertension and arrhythmias. Bevacizumab-induced cardiotoxicity is associated with endoplasmic reticulum stress, mitochondrial dysfunction, ERK pathway inactivation, microvascular injury and vascular stiffness [42,43]. Although these data show that copeptin might serve as a promising marker for the risk stratification of cardiotoxicity, the results of our study do not show any association between the serum level of copeptin and treatment outcomes. This might be due to the relatively small group of patients included in the study or the short treatment evaluation of only six months. Cardiotoxic effects may manifest later in the evolution of the disease. Copeptin-related results can also be influenced by the associated chemotherapy regimen. Although bevacizumab was administered together with fluoropyrimidines, there were patients who received oral chemotherapy with capecitabine, a precursor of the 5-fluorouracil fraction, combined with oxaliplatin, irinotecan or administered as monotherapy.

Recent data show that the Ang-Tie2 signaling is important in vascular development, and it plays an indispensable role in the recruitment of supporting wall cells, in the remodeling and maturation of the embryo vessels, as well as during the angiogenesis process [20,21,44,45]. Ang-2 might have a role in destabilizing the interactions between the endothelial cells and perivascular cells, stimulating vascular regression, but, in the presence of VEGF-A, Ang-2 stimulates angiogenesis. Therefore, the activation Ang-Tie2 acts as an angiogenic modulator during tumor progression and metastasis. The results of the study by Jayson et al. showed that Tie2 may function as a biomarker for bevacizumab in metastatic CRC patients. Following treatment with bevacizumab, a correlation was observed between Ang-2, Tie2, and endothelial transfer coefficient (K^trans^), a tumor vascular imaging biomarker, suggesting that Tie2 originates from the tumor vasculature. Moreover, sustained decreases in Tie2 levels are correlated with improved PFS [46]. These results are not supported by our study, in which serum Tie2 levels were not associated with OS or PFS; this is potentially due to the different study methodologies. Firstly, the measurement of biomarkers was performed differently. In our study, the samples were collected just before initiating treatment, and then after one and six months of therapy. In the study by Jayson et al., markers were measured at baseline; on days 3, 15, 22; at week 6; at 6 months; and at the time of disease progression. Secondly, the first treatment administered in their study included bevacizumab as monotherapy, and the FOLFOX protocol chemotherapy was added after only 2 weeks. In our study, both bevacizumab and chemotherapy were administered in combination right from the first cycle. The attending physician, depending on the patient’s characteristics, varying between oxaliplatin-based, irinotecan-based or fluoropyrimidine monotherapy, established the associated chemotherapy regimen. Thus, the potential correlations noted by Jayson et al. between circulating and vascular imaging biomarkers might result from including bevacizumab in monotherapy 2 weeks prior to chemotherapy administration.

As we demonstrated in a previous study [47], therapy-induced proteinuria and anemia are prognostic factors for patients with CRC following treatment with bevacizumab and chemotherapy. These results are also supported by the current analysis. The identification of an episode of anemia during treatment, regardless of grade, was associated with lower OS. A Cox analysis showed the presence of anemia-influenced OS, regardless of CypA. Although patients with proteinuria had a better OS (47 versus 27 months), the difference was not statistically significant in the present study (*p* = 0.054; Figure 3E); however, according to the Cox analysis, proteinuria is an important prognostic factor (Table 4).

A retrospective analysis by Dionísio de Sousa et al. showed that over half (51.9%) of the 79 patients with metastatic CRC treated with first-line bevacizumab and chemotherapy developed grade 2 or 3 hypertension. From these, 73.2% achieved a tumor response (complete or partial). In the group without hypertension during treatment, only 18.4% achieved a complete or partial response, and 63.2% had a stable disease. Bevacizumab-induced hypertension was an independent predictive factor for PFS but not for OS (33 versus 21 months, *p* = 0.114) [48]. In another study, the onset of hypertension within 3 months of the initiation of bevacizumab treatment was associated with improved OS in patients with CRC. From the 101 patients included, 56% developed hypertension of grade ≥1; 26% of them were normotensive undergoing antihypertensive treatments, but experienced episodes of blood pressure increase after initiation of bevacizumab. OS in the hypertension group was 25.8 months, compared with 11.7 months in the other group of normotensive patients. The disease-free interval was also longer in the first group: 10.5 months versus 5.3 months, as were the response rates: 30% versus 20% [49]. Those results are in accordance with our data in the sense that patients who experienced increases in blood pressure had a better PFS and OS, but only the difference in PFS was statistically significant. 

Our research is limited to a cohort of 56 patients; thus, more extensive validation studies are needed to elucidate the additional roles of CypA in metastatic CRC. Importantly, since the study began before and comprised the period during the COVID-19 pandemic, the relatively smaller size of our cohort is also a reflection of the lower addressability of CRC patients to health care systems and compliance to therapy caused by pandemic restrictions in our country. Another limitation is that we did not consider additional prognostic factors known to be associated with reduced overall survival, such as the presence of KRAS or BRAF mutations [5].

### Clinical Implications

The results of our study provide information on the dynamics of serum CypA levels and their association with bevacizumab as antiangiogenic therapy in metastatic CRC. To the best of our knowledge, this is the first study that investigates the prognostic and predictive role of CypA for chemotherapy associated with bevacizumab. These data could serve as a basis for more extensive future studies, not just limited to colorectal cancer but also applicable to other types of cancer where antiangiogenic therapy is utilized. 

As our data show that high CypA levels, both at baseline and after one month of treatment, correlate with lower OS, we believe that the results of our study directly impact clinical practice by identifying patients with a poorer prognosis that require more aggressive treatment approaches, such as a combination of triple chemotherapy.

With more extensive validation studies, CypA levels could assist in stratifying patients with metastatic CRC for targeted therapy. Within the current study, patients exhibiting low CypA levels after 6 months of treatment with bevacizumab and chemotherapy demonstrated improved PFS, suggesting a potential benefit from therapy, compared to those with high CypA levels. In addition, CypA may play a therapeutic role; therefore, targeting CypA could be a promising strategy in increasing the efficacy of antiangiogenic therapy combined with chemotherapy.

## 5. Conclusions

The findings of our research indicate that—in addition to the number of lymph node tumor invasions, number and location of metastases, treatment-induced anemia and proteinuria—low values of CypA, both at baseline and after one month of treatment with bevacizumab and chemotherapy in metastatic CRC patients, represent an independent prognostic factor for OS and are correlated with a better prognosis. Importantly, copeptin and Tie2 were not associated with OS or PFS in our study.

## Figures and Tables

**Table 1:** Patient and disease characteristics.

Characteristic	Frequency	Percent
Median age, years (range)	61 (37–82)	
Gender		
Male	30	54
Female	26	46
Primary tumor location		
Descending colon	41	73
Ascending colon	15	27
Stage at diagnosis		
Metastatic	44	79
Non-metastatic	12	21
Primary tumor resection	40	71
RAS status		
Wild type	17	31
Mutant	36	64
Not tested	3	5
Associated chemotherapy		
Oxaliplatin-based	37	66
Irinotecan-based	16	29
Fluorouracil/Capecitabine-based	3	5
Preexisting arterial hypertension	27	48
High blood pressure during treatment	40	71
Number and location of metastases		
Liver	24	43
Lung	4	7
Peritoneal	4	7
Multiple	24	43
Tumor response		
CR ^1^	2	3.6
PR ^2^	11	19.6
SD ^3^	25	44.6
PD ^4^	18	32.1

^1^ CR—complete response; ^2^ PR—partial response; ^3^ SD—stable disease; ^4^ PD—progressive disease.

**Table 2:** Adverse events of treatment with bevacizumab and chemotherapy ^1^.

Event	All Grades *n* (%)	Grades ≥ 3 *n* (%)
Any	54 (96.4)	13 (23.2)
Proteinuria	30 (53.6)	*
Anemia	20 (35.7)	5 (9)
Neutropenia	26 (46.4)	7 (12.5)
Thrombocytopenia	19 (34)	0
Neurological toxicity ^2^	25 (44.7)	1 (1.8)
Liver toxicity ^3^	17 (30.4)	0

^1^ Classified according to Common Terminology Criteria for Adverse Events v4.0. * Evaluation of proteinuria was qualitative only. ^2^ Neurological toxicity refers to secondary peripheral neuropathy. ^3^ Liver toxicity was assessed by ALT, AST and GGT values.

**Table 3:** Serum concentrations and median values of Cyclophilin A, copeptin and Tie2 in colorectal cancer patients.

Biomarker	Baseline Levels (Median, Min–Max)	After One Month Treatment Levels (Median, Min–Max)	After Six Months Treatment Levels (Median, Min–Max)
Cyclophilin A (ng/mL)	54.65	29.38	114.92
	(1.54–168.61)	(1.05–400.03)	(0.72–432.71)
copeptin (pg/mL)	492.54	884.35	950.42
	(129.67–1183.92)	(206.15–6740.80)	(402.93–4308.60)
Tie2 (ng/mL)	0.56	0.62	0.55
	(0.44–12.34)	(0.46–12.54)	(0.39–8.88)

**Table 4:** Associations between CypA levels, baseline, post-treatment factors and OS for patients with metastatic CRC, using multivariate Cox analysis.

Factor	*p*	HR	95% Confidence Interval
**CypA at baseline adjusted for baseline factors:**			
CEA	0.929	-	-
CA 19-9	0.643	-	-
LDH	0.711	-	-
Number of metastases	0.224	-	-
Resection of the primary tumor	0.181	-	-
Number of lymph node invasion	0.010	1.981	1.182–3.321
CypA at baseline	0.016	0.424	0.210–0.855
**CypA at baseline adjusted for post-treatment factors:**			
Thrombocytopenia	0.568	-	-
Neutropenia	0.755	-	-
Liver toxicity	0.991	-	-
Anemia	0.002	0.317	0.151–0.666
Proteinuria	0.026	2.197	1.100–4.390
CypA at baseline	0.004	0.330	0.156–0.694
**CypA after one month of treatment adjusted for baseline factors:**			
CEA	0.096	-	-
CA 19-9	0.184	-	-
LDH	0.735	-	-
Number of metastases	0.109	-	-
Resection of the primary tumor	0.068	-	-
Number of lymph node invasion	0.017	1.829	1.113–3.005
CypA after one month of treatment	0.101	-	-
**CypA after one month of treatment adjusted for post-treatment factors:**			
Thrombocytopenia	0.521	-	-
Neutropenia	0.900	-	-
Liver toxicity	0.923	-	-
Anemia	0.008	0.386	0.190–0.782
Proteinuria	0.019	2.312	1.146–4.665
CypA after one month of treatment	0.013	0.406	0.200–0.828

**Table 5:** Associations between CypA levels, treatment-induced hypertension and PFS for patients with metastatic CRC, using multivariate Cox analysis.

Factor	*p*	HR	95% Confidence Interval
CypA after 6 months of treatment	0.011	0.461	0.253–0.840
Treatment-induced hypertension	0.001	3.315	1.636–6.716

## Data Availability

The data presented in the study are available on request from the corresponding authors and can be shared with the journal if needed.

## Supplementary Materials

The following supporting information can be downloaded at: https://www.mdpi.com/article/10.3390/cancers16020385/s1, Figure S1: Kaplan–Meier survival curves for patients with low or high copeptin values; Figure S2: Kaplan–Meier survival curves for patients with low or high Tie2 values; Table S1: The REMARK checklist. Reference [50] is cited in the Supplementary Materials.
