# Supplementary material for: Cyclophilin A: An Independent Prognostic Factor for Survival in Patients with Metastatic Colorectal Cancer Treated with Bevacizumab and Chemotherapy
Source: Cancers (Basel). 2024 Jan 16;16(2):385. doi: 10.3390/cancers16020385 (PMC10814009; doi:10.3390/cancers16020385)
Supplement: Supplementary file 1 [file cancers-16-00385-s001.zip › Supplementary figures.pdf]

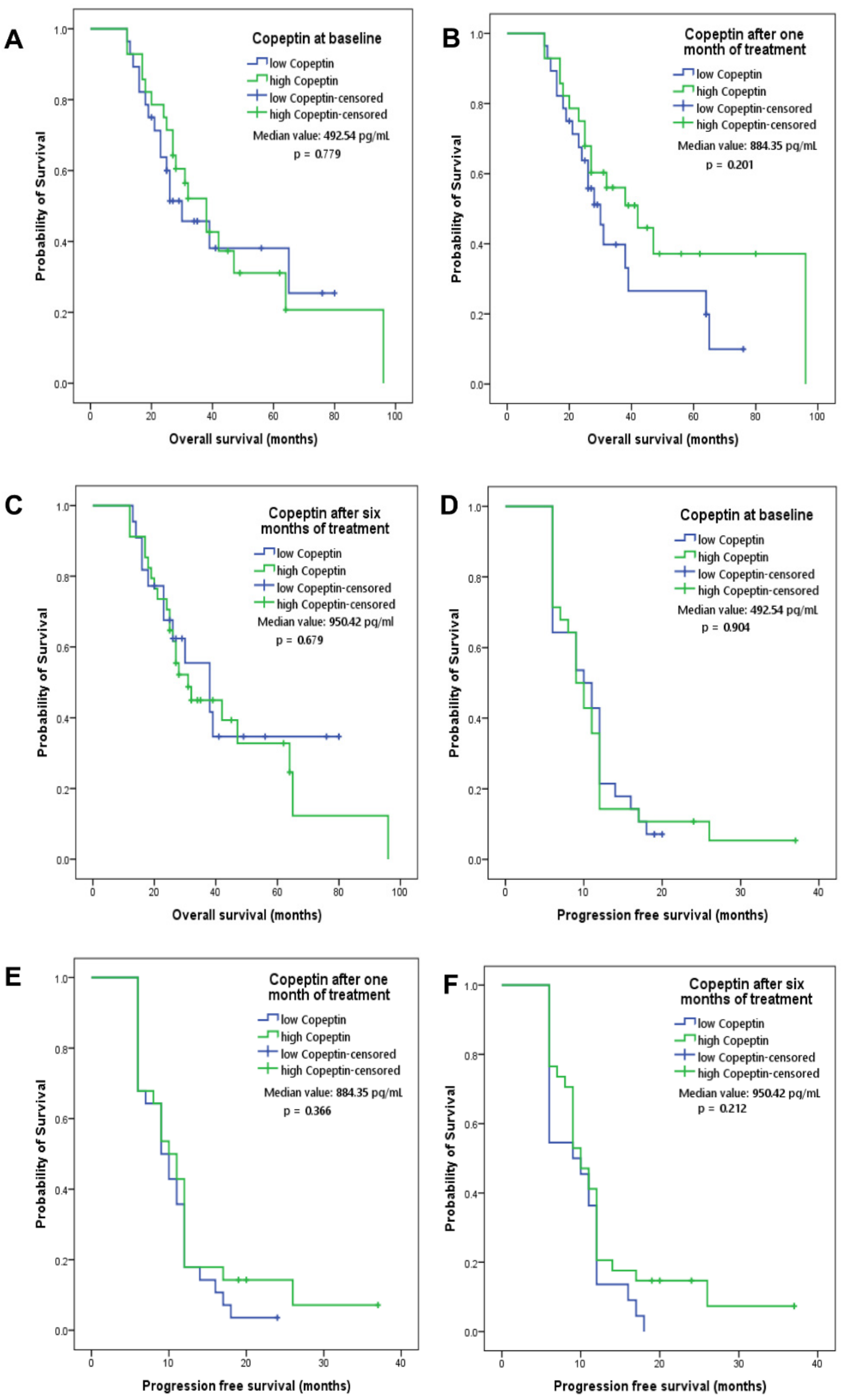

Figure S1. Kaplan–Meier curves of overall survival for patients with low or high Copeptin values at (A) baseline, (B) after one month, and (C) after six months of treatment. Kaplan–Meier curves of progression free survival for patients with low or high Copeptin values at (D) baseline, (E) after one month, and (F) after six months of treatment.

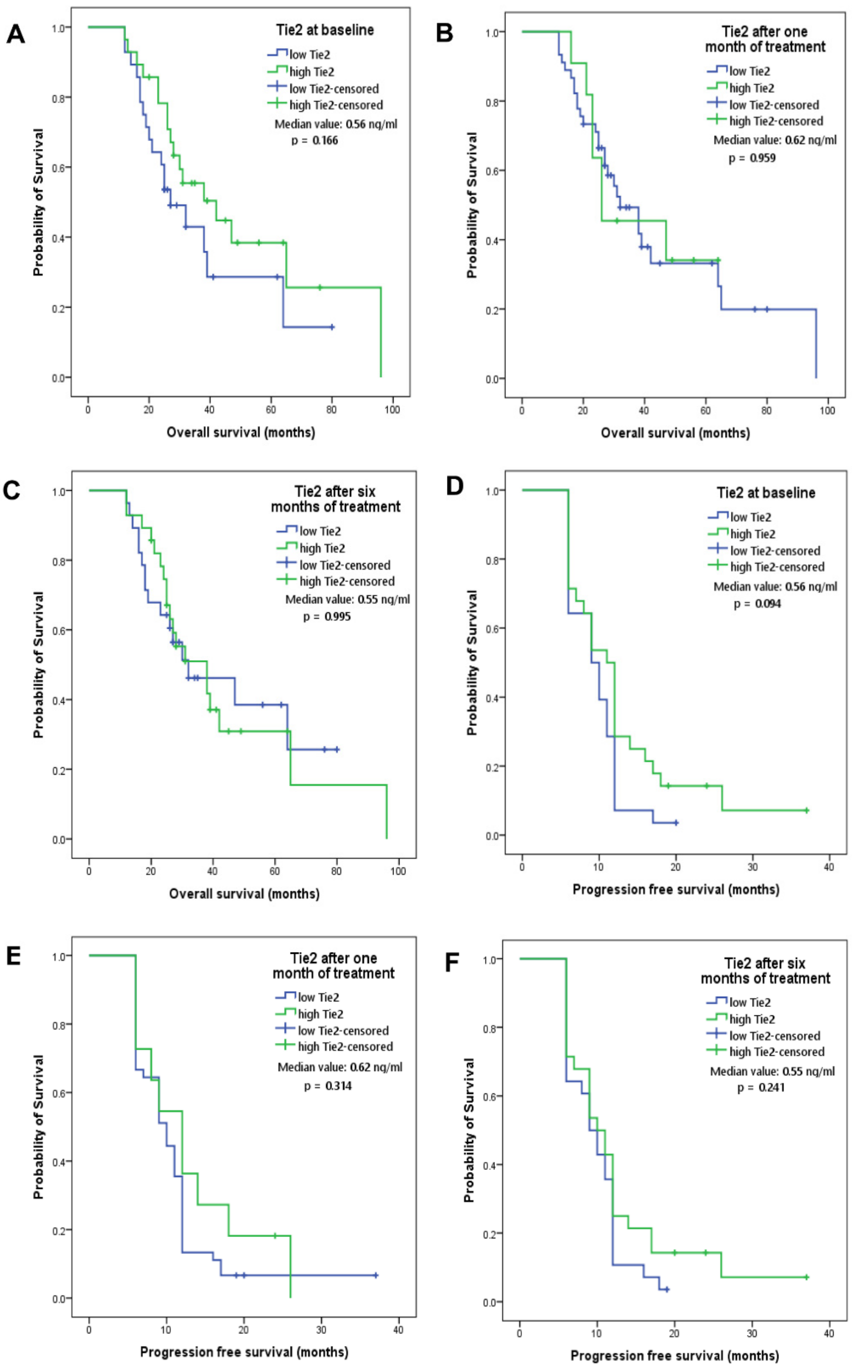

Figure S2. Kaplan–Meier curves of overall survival for patients with low or high Tie2 values at (A) baseline, (B) after one month, and (C) after six months of treatment. Kaplan–Meier curves of progression free survival for patients with low or high Tie2 values at (D) baseline, (E) after one month, and (F) after six months of treatment.
